# Mutations in LRRK2 impair NF-κB pathway in iPSC-derived neurons

**DOI:** 10.1186/s12974-016-0761-x

**Published:** 2016-11-18

**Authors:** Rakel López de Maturana, Valérie Lang, Amaia Zubiarrain, Amaya Sousa, Nerea Vázquez, Ana Gorostidi, Julio Águila, Adolfo López de Munain, Manuel Rodríguez, Rosario Sánchez-Pernaute

**Affiliations:** 1 Laboratory of Stem Cells and Neural Repair, Inbiomed, Paseo Mikeletegi, 81, E-20009 San Sebastian, Spain; 2 Laboratory of Ubiquitylation and Cancer Molecular Biology, Inbiomed, San Sebastian, Spain; 3 Genomics Platform and Neuroscience Area, Biodonostia Research Institute, San Sebastian, Spain; 4 Neurology Department, Donostia Universitary Hospital, Neuroscience Area, Instituto Biodonostia, San Sebastián, Spain; 5 Center for Biomedical Research Network in Neurodegenerative Diseases (CIBERNED), Institute Carlos III, Ministry of Economy and Competitiveness, Madrid, Spain; 6 Department of Neurosciences, University of the Basque Country, UPV/EHU, San Sebastian, Spain

**Keywords:** Parkinson’s disease, LRRK2, Inflammation, iPSCs, NF-κB, α-Synuclein

## Abstract

**Background:**

Mutations in leucine-rich repeat kinase 2 (LRRK2) contribute to both familial and idiopathic forms of Parkinson’s disease (PD). Neuroinflammation is a key event in neurodegeneration and aging, and there is mounting evidence of LRRK2 involvement in inflammatory pathways. In a previous study, we described an alteration of the inflammatory response in dermal fibroblasts from PD patients expressing the G2019S and R1441G mutations in LRRK2.

**Methods:**

Taking advantage of cellular reprogramming, we generated induced pluripotent stem cell (iPSC) lines and neurons thereafter, harboring LRRK2^G2019S^ and LRRK2^R1441G^ mutations. We used gene silencing and functional reporter assays to characterize the effect of the mutations. We examined the temporal profile of TNFα-induced changes in proteins of the NF-κB pathway and optimized western blot analysis to capture α-synuclein dynamics. The effects of the mutations and interventions were analyzed by two-way ANOVA tests with respect to corresponding controls.

**Results:**

LRRK2 silencing decreased α-synuclein protein levels in mutated neurons and modified NF-κB transcriptional targets, such as *PTGS2* (*COX-2*) and *TNFAIP3* (*A20*). We next tested whether NF-κB and α-synuclein pathways converged and found that TNFα modulated α-synuclein levels, although we could not detect an effect of LRRK2 mutations, partly because of the individual variability. Nevertheless, we confirmed NF-κB dysregulation in mutated neurons, as shown by a protracted recovery of IκBα and a clear impairment in p65 nuclear translocation in the LRRK2 mutants.

**Conclusions:**

Altogether, our results show that LRRK2 mutations affect α-synuclein regulation and impair NF-κB canonical signaling in iPSC-derived neurons. TNFα modulated α-synuclein proteostasis but was not modified by the LRRK2 mutations in this paradigm. These results strengthen the link between LRRK2 and the innate immunity system underscoring the involvement of inflammatory pathways in the neurodegenerative process in PD.

**Electronic supplementary material:**

The online version of this article (doi:10.1186/s12974-016-0761-x) contains supplementary material, which is available to authorized users.

## Background

Parkinson’s disease (PD) is a neurodegenerative disorder characterized by a progressive and relatively selective death of dopamine (DA) neurons within the substantia nigra of the midbrain [1]. The neuropathological hallmark of PD is the Lewy body fibrillar aggregates in which α-synuclein is the major constituent [2]. The great majority of PD cases are sporadic, with only 5–10% being familial. Mutations in the leucine-rich repeat kinase 2 (*LRRK2*, *PARK8*) gene are the most common cause of monogenic PD [3]. Furthermore, both common and uncommon variants are associated with an increase odd risk in GWAS analyses [4]. The precise physiological function of LRRK2 has yet to be defined due to its involvement in multiple pathways, but we and others have proposed an active role in the immune response (reviewed in [5]). Indeed, in a previous study, we demonstrated a defective NF-κB activation in response to a pro-inflammatory stimulus in dermal fibroblasts from PD patients [6]. NF-κB activation is responsible for the intracellular regulation of age-related inflammation which appears to play a major role in neurodegeneration.

LRRK2 is a large multi-domain protein with two enzymatic activities, a serine/threonine kinase and a ROC (Ras of complex)-GTPase [3]. The G2019S substitution in the kinase activation loop is by far the most common pathogenic mutation and increases the kinase activity [7, 8]. The R1441G/C/H/S substitutions in the ROC-GTPase domain generally result in lower GTPase activity, with more inconsistent effects on kinase activity [3]. Despite these differences, most pathogenic mutants display an increase in the (auto)phosphorylation at Ser1292 [9] and also in the phosphorylation of at least one other substrate, the Rab GTPases [10]. Unraveling a common mechanism for all LRRK2 mutations is critical for understanding LRRK2 role in PD pathogenesis.

Increasing experimental evidence underscores the involvement of LRRK2 in the inflammatory response, supported also by the robust LRRK2 expression in immune cells, including peripheral monocytes and macrophages, and in primary microglia (reviewed in [5]). The link to the innate immune response is further reinforced by the genetic association of LRRK2 with susceptibility to inflammatory bowel disorder [11] and leprosy [12, 13]. Moreover, the expression of LRRK2 is modulated by immune cell-specific signals, like IFNγ and toll-like receptor (TLR) agonists [14–16].

In order to examine inflammatory responses in a disease-relevant context, we extended our previous work on patients’ fibroblasts harboring LRRK2^G2019S^ and LRRK2^R1441G^ mutations by reprogramming the cells and using neurons derived from the induced pluripotent stem cells (iPSCs). In this cellular model, which preserves the endogenous (and regulated) expression of LRRK2, we examined the effect of the mutations on α-synuclein and TNFα-induced NF-κB activation, with the hypothesis that inflammatory stimuli can modulate α-synuclein proteostasis.

## Methods

### Reprogramming and generation of iPSC lines

The experimental protocol was approved by the Ethical Committee at Hospital Donostia (San Sebastian, Spain), and all procedures adhered to the internal and EU guidelines for research involving derivation of pluripotent cell lines. All subjects gave informed consent for the study using forms approved by the Ethical Committee on the Use of Human Subjects in Research at Hospital Donostia and Onkologikoa Hospital, both in San Sebastian, Spain. Generation of iPSC lines was approved by the Advisory Committee for Human Tissue and Cell Donation and Use, Instituto Carlos III (ISCIII), Madrid, Spain. All procedures were done in accordance with institutional guidelines, and the cell lines have been deposited at the Banco Nacional de Lineas Celulares (BNLC, ISCIII) following the Spanish legislation.

Skin fibroblast cultures from PD patients with mutations in LRRK2 and matched healthy subjects have been previously characterized in our laboratory [6]. We included samples from four men and two women, with a median age of 62.5 ± 13.9 years. Dermal fibroblasts were cultivated as described previously [6].

**Fig. 1:**
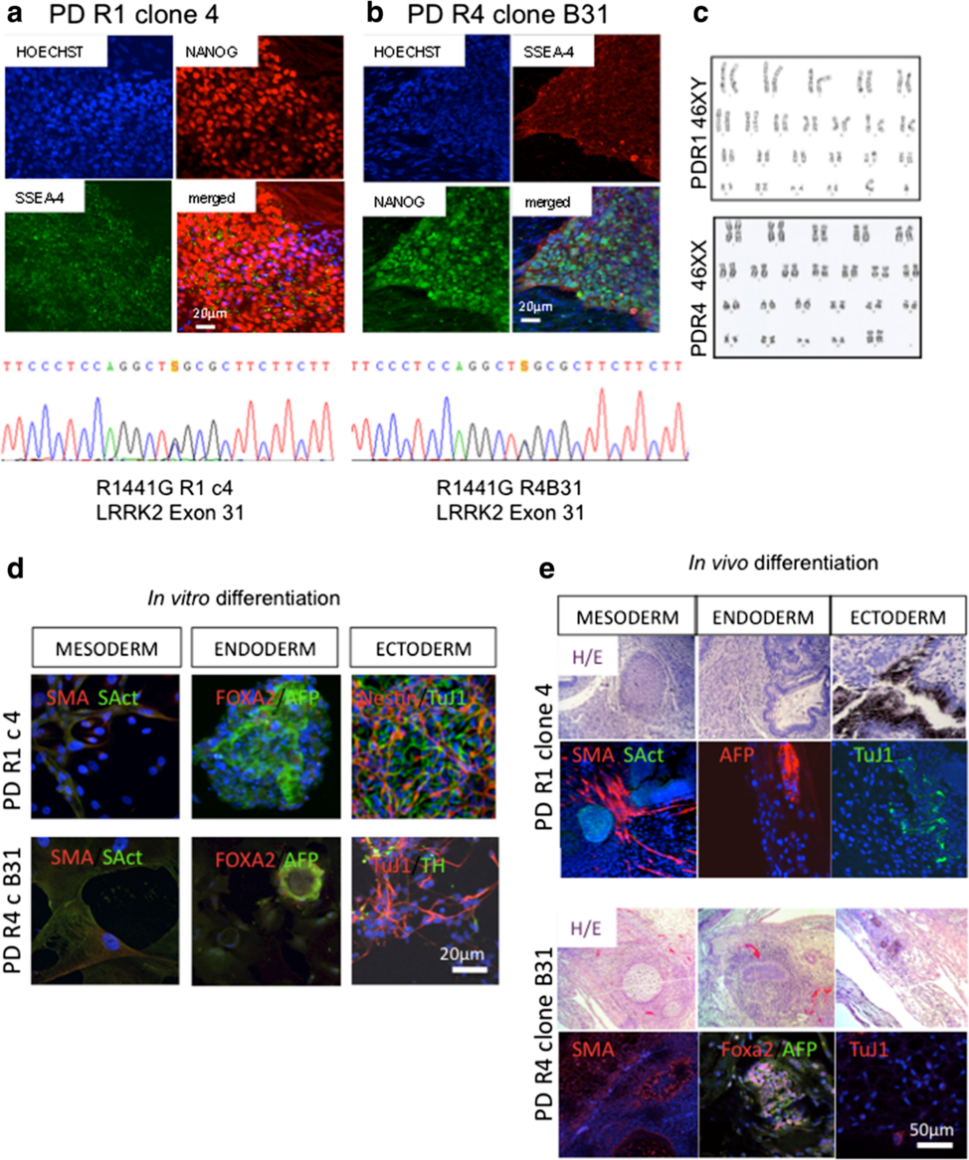
Characterization of pluripotency of the LRRK2^R1441G^ mutant iPSC lines (PD-R1 and PD-R4). **a**, **b** Reprogrammed cells formed compact uniform colonies that showed robust and uniform expression of typical pluripotent markers such as NANOG and embryonic stage-specific antigen 4 (SSEA-4) by immunofluorescence. Sequence analysis of exon 31 confirmed the presence of the point mutation in the clones selected for this study. **c** G-band karyotypes for the selected clones. Pluripotency was confirmed **d** in vitro by embryoid body formation and trilineage differentiation, analyzed by immunofluorescence and **e** in vivo by formation of teratomas in NOD-SCID mice that showed cells corresponding to the three germ layers. *SMA* smooth muscle actin, *SAct* sarcomeric actin, *AFP* alpha fetoprotein, *TUJ1* βIII-tubulin, *TH* tyrosine hydroxylase, *H/E* hematoxylin/eosin. *Scale bars* are indicated in each panel

For this study, we reprogrammed fibroblasts from two LRRK2^G2019S^ and two LRRK2^R1441G^ different patients. We used lentiviral vectors containing c-Myc, Oct-4, Sox-2, and Klf-4 as previously reported [17], and iPS cell lines were characterized following standard procedures defined by the BNLC in accordance with international guidelines. Results for the two LRRK2^R1441G^ (that have not been previously reported in the literature) are shown in Fig. 1. Controls included a human embryonic stem cell line (H9) to control for a possible effect of the lentiviral reprogramming procedure and iPSC lines from healthy individuals, previously generated in our laboratory [17].

### Maintenance of iPSCs and differentiation to DA neurons

iPSCs were cultured and differentiated as described in [17] with minor modifications. iPSCs were maintained on irradiated human foreskin fibroblasts (HFF-1, SCRC-1041, ATCC) in hES cell medium made of knockout-DMEM (10829-018, Invitrogen), supplemented with 2 mM Glutamax (#3505003, Invitrogen), 50 nM β-mercaptoethanol (31350-010, Invitrogen), 1× non-essential amino acids (M7145, Sigma), 20% knockout serum (KSR10828028, Invitrogen), 50 U/ml penicillin, 50 μg/ml streptomycin, and 10 ng/ml FGF2 (100-18B, PeproTech). iPS cell colonies were passaged manually once a week.

**Fig. 2:**
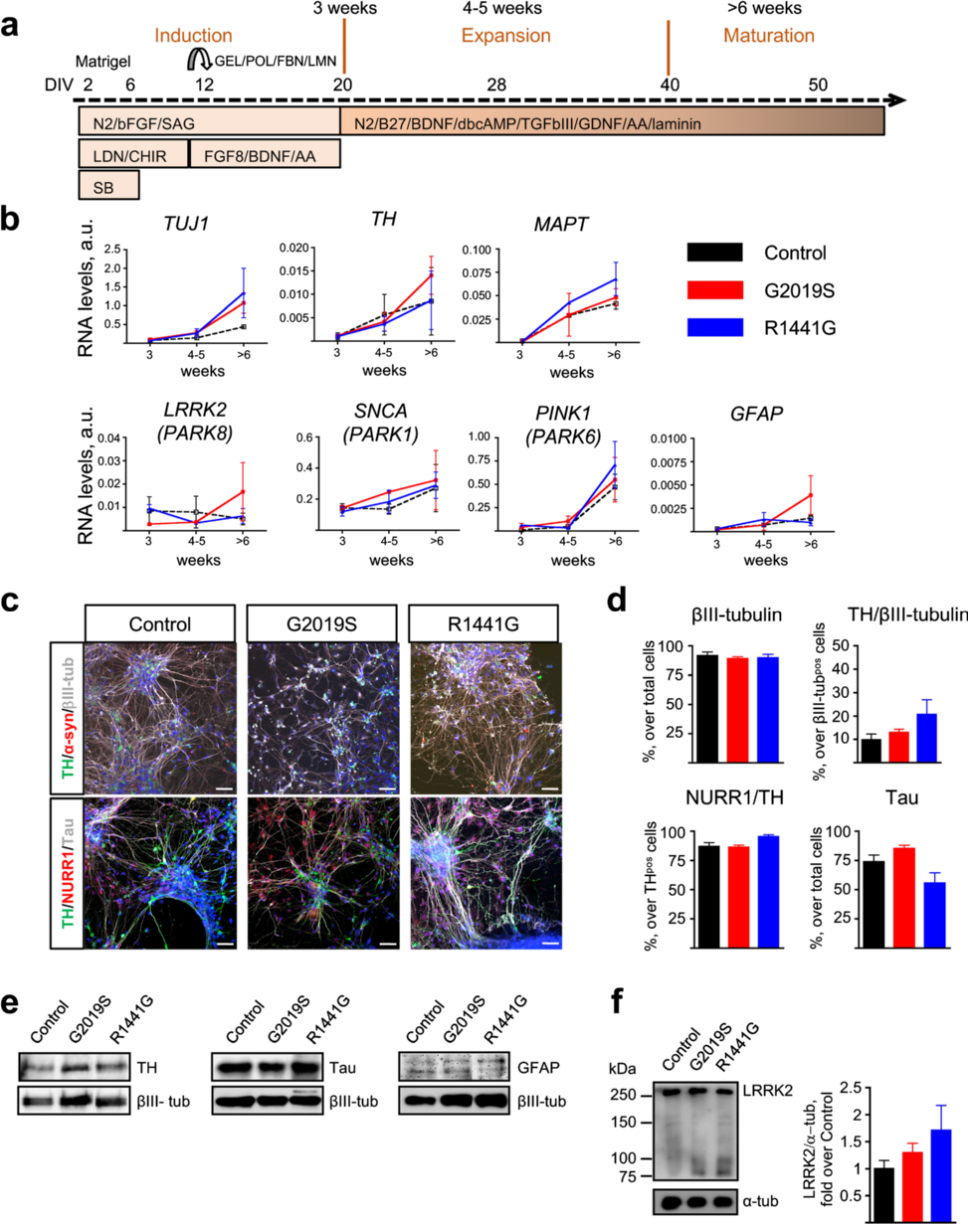
Characterization of iPSC-derived DA neurons with LRRK2 mutations. **a** Diagram showing the DA differentiation protocol used for neural induction of human iPSC lines. **b** Temporal gene expression analyzed by qRT-PCR at three time points: induction (3 weeks), expansion (4–5 weeks), and maturation (>6 weeks). Each point represents the mean ± SEM of at least two independent differentiation experiments. **c** Representative images of mature neuronal cultures showing expression of neuronal (βIII-tubulin, Tau, and α-synuclein) and dopaminergic (TH, NURR1) markers. Nuclei were counterstained with Hoechst. *Scale bars*: 50 μm. **d** Quantification of immunostainings. Data are represented as mean ± SEM of counts from at least two different lines for each genotype. **e** Representative western blot analyses of TH, Tau, and GFAP with βIII-tubulin as loading control in iPSC-derived mature neurons. **f** Representative immunoblots and quantification of LRRK2 expression in mature neuronal cultures. α-tubulin was the loading control and data were normalized to control WT neurons. *Bars* represent the mean ± SEM of at least two different lines per genotype. *DIV* days in vitro, *GEL* gelatin, *POL* poly-ornithine, *FBN* fibronectin, *LMN* laminin, *N2* N2 supplement, *bFGF* basic fibroblast growth factor, *SAG* smoothened agonist, *LDN* LDN-193189, *CHIR* CHIR99021, *SB* SB431542, *BDNF* brain-derived neurotrophic factor, *AA* ascorbic acid, *B27* B27 supplement, *dbcAMP* dibutyryl cyclic adenosine monophosphate, *TGFβIII* transforming growth factor βIII, *GDNF* glial derived neurotrophic factor. See Additional file 2 for uncropped blots

The protocol used for DA induction and differentiation is shown schematically in Fig. 2a. Undifferentiated cells were plated on Matrigel in hES cell medium (D0), and 1 day later (D1), half of the hES cell medium was replaced with DMEM-F12 supplemented with 1× N2 supplement (#17502048, Invitrogen) and 10 ng/ml FGF2. Neural induction was started at D2 by adding 10 μM SB431542 (#1614, Tocris) and 100 nM LDN-193189 (130-096-226, Miltenyi Biotech). For the induction of DA phenotype, 500 nM SAG (smoothen agonist, #566660, Millipore), and 0.5 nM CHIR 99021 (#13122, Cayman Chemical) were added to the medium. On D12, neural rosettes were mechanically passaged onto 0.1% gelatine (G1393; Sigma), 15 μg/ml polyornithine, 1 μg/ml fibronectin, and 1 μg/ml laminin-coated plates and expanded at high cell densities. At this time, LDN and CHIR 99021 were removed from the medium; SAG was reduced to 20 nM, and 100 ng/ml FGF8 was added. At D14, the medium was also supplemented with 20 ng/ml BDNF and 200 μM ascorbic acid (BASF medium). Subsequent passages of neural progenitors were done using Accutase® (Sigma-Aldrich®). On ∼D20, BASF medium was replaced with BCT-GA medium, composed of neurobasal medium with 1× N2 supplement, 1× B27 supplement (17504-044, Invitrogen), 2 mM Glutamax, 20 ng/ml BDNF, 10 ng/ml GDNF, 200 μM ascorbic acid, 0.5 mM dibutyryl-cAMP, 1 ng/ml TGF-β III, and 1 μg/ml laminin. As shown in Fig. 2a, we defined three stages: induction stage, weeks 1–3 (D0–D20); expansion stage, weeks 4–5 (D28-D35); and maturation stage, week 6–onwards (>D40). All the experiments were carried out in the maturation stage.

To evaluate the NF-κB activity, cells were stimulated by the addition of TNFα (#210-TA-005, R&D Systems), with 10 and 15 ng/ml as indicated in the “Results” section.

### LRRK2 gene knockdown

Endogenous LRRK2 expression was silenced as previously described [6] using the MISSION® shLRRK2 vector (TRCN0000358257, SIGMA) with a multiplicity of infection (MOI) of 8. As a control, cells were transduced with the empty vector (mock control). The viral supernatant was removed 18 h later and replaced with fresh growth medium. The following experiments were carried out 5 days after transduction.

### Western blotting

For whole-cell lysate preparation, neurons were harvested using Accutase®, washed in PBS and lysed in RIPA lysis buffer (50 mM Tris-HCl pH 8, 150 mM NaCl, 1 mM EDTA, 1% NP-40, 0.1% SDS, 0.25% sodium deoxycholate) with 1 mM sodium orthovanadate, 1 mM NaF, and a protease inhibitor cocktail (Roche). For probing α-synuclein, the cells were washed with PBS and lysed in a modified RIPA buffer (50 mM Tris-HCl pH 8, 150 mM NaCl, 1 mM EDTA, 1% Triton X-100, 0.5% SDS, 0.5% sodium deoxycholate, 5% glycerol). Lysates were briefly sonicated, clarified by centrifugation at 15,000 rpm for 15 min and finally resolved by SDS-PAGE. Immunoprecipitation was performed using Protein-G cross-linked with the anti-p62 antibody (P0067, Sigma). For the kinetic experiments, the cells were stimulated with TNFα, washed with PBS 1×, and lysed in SDS-PAGE loading buffer (2% SDS, 5% 2-mercaptoethanol, 10% glycerol, 0,1% bromophenol blue, 62.5 mM Tris-HCl, pH 6.8). After SDS-PAGE, the proteins were transferred to polyvinylidene difluoride (PVDF) membrane (Immobilon-P, Merck-Millipore). The following antibodies were used at a 1:1000 dilution: LRRK2 (NB110-58771, Novus Biologicals), p62 (P0067, Sigma), Sam68 (C-20, #sc-333, Santa Cruz Biotechnology), IκBα (L35A5, #4814, Cell Signalling), α-synuclein (MA1-12874, Thermo Scientific), Tau (T46, #136400, Invitrogen), βIII-tubulin (mms-435p, Covance), and TH (P60101-0, Pel Freez Biologicals); α-tubulin (DM1A, #3873, Cell Signalling Technology®) was used at 1:5000 dilution. A horseradish peroxidase-conjugated IgG (GE Healthcare) was employed as a secondary antibody. Visualization of HRP-labeled proteins was performed using enzyme-linked chemiluminescence (ThermoFisher Scientific) either detected on X-ray films or directly with a digital CCD camera (Versadoc Imager, Bio-Rad). Bands were quantified by densitometry relative to the corresponding loading control using ImageJ (NIH).

### Immunofluorescence

Cells cultured on sterile glass cover slips were fixed with 4% paraformaldehyde for 10 min at room temperature. The cells were then permeabilized and blocked with 10% donkey serum in 0.1% Triton X-100/PBS for 45 min. Primary antibody incubation was performed overnight at 4 °C, using the following antibodies diluted in PBS as indicated: Nanog (1:100, R&D), SSEA-4 (1:100, Hybridoma Bank, U.IOWA), SMA (1:400, Sigma), sarcomeric actin (1:400, Sigma), AFP (1:400, DAKO), FOXA2 (1:100, Santa Cruz Biotechnology), Nestin (1:500, Neuromics), p65 (1:100, sc-372, Santa Cruz), TH (1:1000, P60101-0, Pel Freez Biologicals), α-synuclein (1:100, MA1-12874, Thermo Scientific), Tau (1:200, T46, #136400, Invitrogen), βIII-tubulin (1:1000, prb-435p-100, Covance), NURR1 (1:200, E-20, sc-990, Santa Cruz), and GFAP (1:500, Z0334, Dako). Next, the cells were washed with 10% donkey serum/PBS and incubated for 1 h with Alexa fluorochrome-conjugated secondary antibodies diluted in PBS. After final washes with 0.1× PBS, the cover slips were mounted using ProLong® antifade reagent (Molecular Probes®, Life Technologies). Images were acquired with a Zeiss LSM510 confocal microscope and analyzed using ImageJ (1.49, NIH). For quantification of neuronal markers, tile images (1272 × 1272 μm) were acquired at a ×40 magnification and at least 1000 cells were counted for each cell line. For the evaluation of nuclear p65 translocation, images were randomly acquired at ×63 magnification, and between 150 and 300 cells were scored for each condition.

### Real-time RT-PCR

Total RNA was extracted using Trizol^®^ total RNA isolation reagent (Gibco®, Life Technologies), followed by the RNeasy Qiaprep (Qiagen) per manufacturer’s protocol. RNA concentration was quantified using a NanoDrop Spectrophotometer (NanoDrop Technologies). cDNA was synthesized from total RNA using random hexamers according to the GeneAmp® RNA PCR Core Kit (Life Technologies) and the High-Capacity cDNA RT kit (Applied Biosystems®, Life Technologies). Real-time PCR was performed using an Applied Biosystems StepOne™ Detection System. Comparative analysis of gene expression levels (ΔΔCt) was carried out using GAPDH as the reference gene. The sequences of the primers are indicated in Additional file 1.

### Luciferase assays

We used the Dual-Luciferase® Reporter (DLR™) Assay System (Promega) to measure the activity of firefly and Renilla luciferases sequentially from a single sample. We used the pNF3ConA-Luc plasmid [6] and the pRL-CMV (#E226A, Promega) for normalization of gene expression. Both plasmids were co-transfected into the neurons by electroporation according to the standard protocols (Neon® Transfection System, Invitrogen™, Life Technologies). Briefly, one million cells were resuspended in 10 μl buffer R containing 1 μg pNF3ConA-Luc, and 100 ng pRL-CMV were subjected to two pulses (1000 V, 10 ms), and re-plated on 12-well plates. Forty-eight hours later, neurons were treated with TNFα for 8 h in neurobasal medium without trophic factor supplementation. Normalized data are expressed as the firefly (NF-κB) divided by the Renilla luciferase activity.

### Data transformation and analysis

Data were analyzed using Prism 4.0 (GraphPad Software). All experiments were performed in at least two different cell lines (from different individuals) for each genotype and in at least two independent differentiations. Bar graphs represent average and SEM. Values were normalized as specified in the figure legends. Comparison between groups was carried out by one-way ANOVA with Dunn’s post-test (one variable) or two-way ANOVA with Bonferroni post-test (two variables). Values of *P* < 0.05 were considered significant.

## Results

### Reprogramming and derivation of LRRK2 iPSC-derived DA neuronal cultures

We generated iPSCs from four PD patients harboring mutations in LRRK2. Two patients carried the G2019S in the kinase domain of the protein and another two patients carried the R1441G mutation in the ROC-GTPase domain (Fig. 1). Several reports have used LRRK2^G2019S^ and LRRK2^R1441C^ iPSC lines, but this is the first study describing iPS cell lines carrying the LRRK2^R1441G^ mutation. There was no effect of the mutations on the reprogramming process, and we obtained pluripotent cell lines that maintained the original mutation, had normal karyotypes, and had potential to generate the three germ layers both in vitro and in vivo (Fig. 1a–e). Cell lines generated for the study have been deposited in the Spanish repository (BNLC) and are available at http://www.isciii.es/ISCIII/es.

We next differentiated iPSCs towards a dopamine phenotype, using a double-SMAD inhibition protocol, illustrated in Fig. 2a. Neuronal markers were first detected by qPCR during the expansion stage and increased gradually with time (Fig. 2b). We analyzed the expression profile of several *PARK* genes (*LRRK2*, *SNCA*, *PINK1*). While *SNCA* and *PINK1* levels increased steadily, *LRRK2* RNA levels were more variable along the differentiation process but were not different between groups. RNA levels of the glial marker *GFAP* were low at all times examined (Fig. 2b).

Immunofluorescence analysis of neurons during the maturation stage demonstrated that 90.4 ± 4.4% of cells were βIII-tubulin-positive and 13.3 ± 6.4% of βIII-tubulin-positive cells were TH-positive. Of these TH-positive cells, 88.3 ± 5.5% co-expressed NURR1 (Fig. 2c, d). These in vitro generated neurons showed a normal developmental expression of specific proteins like the microtubule-associated protein Tau, which was present in 73.5 ± 13.6% of cells in the cultures (Fig. 2c, d). Immunoblot analysis of protein extracts confirmed no differences in either TH or Tau content across genotypes. Consistent with the transcriptional profile, the glial marker GFAP was barely detectable (Fig. 2e, Additional file 2). Importantly, LRRK2 protein expression showed no differences between genotypes at 6 weeks (Fig. 2f).

Taken all analyses together, we concluded that there were no differences in the efficiency of neuronal specification and markers’ expression in these cultures; the low numbers of DA neurons that we obtained in these experiments were not related to the presence of LRRK2 mutations.

### LRRK2 regulates α-synuclein proteostasis

**Fig. 3:**
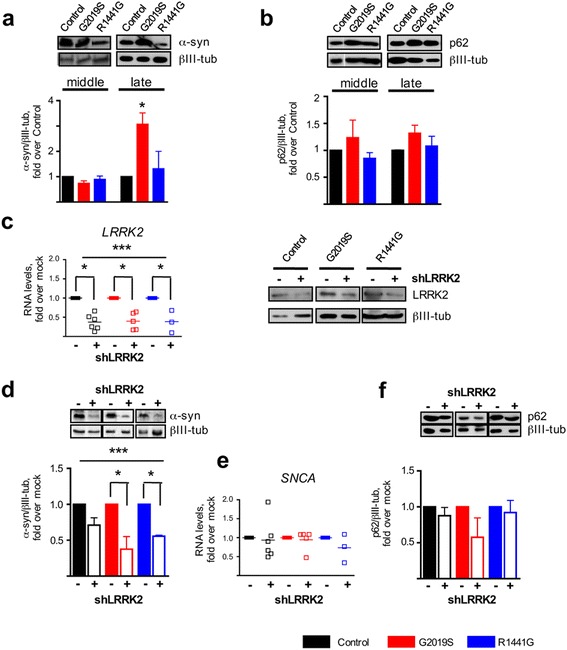
LRRK2 regulation on α-synuclein levels. **a** Western blot analysis of basal α-synuclein and **b** p62 levels in neurons during mid and late differentiations. *Blots* show the α-synuclein soluble monomer of ~15 kDa. *Bars* represent the mean ± SEM of three to six values, including two different cell lines per group. βIII-tubulin was used as loading control (two-way ANOVA, ** P < 0.01, LRRK2^G2019S^ v. control WT, late stage). **c** *LRRK2* RNA levels 5 days after shRNA lentiviral transduction. The empty vector was used as the mock control. *Lines* represent the mean ± SEM of three to four independent silencing experiments. A representative immunoblot analysis of LRRK2 protein levels 5 days after transduction is also shown. βIII-tubulin was the loading control. **d** Immunoblots and corresponding quantification of α-synuclein 5 days after shLRRK2 transduction. *Bars* represent the mean ± SEM of at least three independent silencing experiments. LRRK2 silencing had a significant effect on α-synuclein protein levels (two-way ANOVA, *** P = 0.0002). Bonferroni post hoc test showed that this effect was limited to LRRK2 mutated mature neurons (**P < 0.05 for both LRRK2^G2019S^ and LRRK2^R1441G^). **e** qRT-PCR analysis of *SNCA* after LRRK2 silencing. **f** Immunoblots and corresponding quantification of p62 5 days after shLRRK2 transduction. *Bars* represent the mean ± SEM of at least three independent silencing experiments. See Additional file 3 for uncropped blots

We analyzed the expression of monomeric soluble α-synuclein in our differentiated neurons, since α-synuclein levels have been reported to be higher in LRRK2^G2019S^ neurons [18, 19].We confirmed that in LRRK2^G2019S^ cultures, α-synuclein levels were twofold higher than that in the control wild-type (WT) cultures at the late stage of differentiation (two-way ANOVA, *P* < 0.01, LRRK2^G2019S^ v. WT LRRK2, late stage) (Fig. 3a, Additional file 3). Interestingly, from the point of view of age-related neurodegeneration, this phenotype was only observed in mature neurons (two-way ANOVA, effect of time, *P* < 0.05). Also notably, this appears to be quite specific for LRRK2^G2019S^ neurons, since α-synuclein levels were not increased in the LRKK2^R1441G^ neuronal cultures (Fig. 3a). The autophagic mediator p62, implicated in the removal of α-synuclein, has been reported to be increased in LRRK2^G2019S^ neurons [19]. However, we did not find any differences in p62 basal levels in neurons with either LRRK2 mutation at any stage (Fig. 3b).

To better define the role of LRRK2 in the regulation of these proteins in neurons, we analyzed α-synuclein after silencing endogenous LRRK2 expression. Analysis 5 days after transduction showed an average decrease in *LRRK2* RNA of 60 ± 10%, compared to mock-transduced cells, and a corresponding decrease in LRRK2 protein levels (Fig. 3c). The presence of mutations did not affect silencing efficiency. LRRK2 knockdown significantly reduced α-synuclein protein levels overall, and this effect was driven by the marked decrease observed in the mutant neurons (two-way ANOVA, *P* < 0.001; Bonferroni post-test, *P* < 0.05 for both LRRK2^G2019S^ and LRRK2^R1441G^) (Fig. 3d). This result shows that mutations in LRRK2 affect α-synuclein regulation in neurons. Despite this large effect on protein levels, *SNCA* RNA did not change in LRRK2 silenced neurons, indicating that the regulation is not taking place at the transcriptional level (Fig. 3d). Therefore, we next examined p62 in LRRK2-silenced neurons, and we did not observe changes in its protein levels, concluding that LRRK2 modulation of α-synuclein is independent of p62, at least in these conditions (Fig. 3e).

### Transcriptional effects of LRRK2

**Fig. 4:**
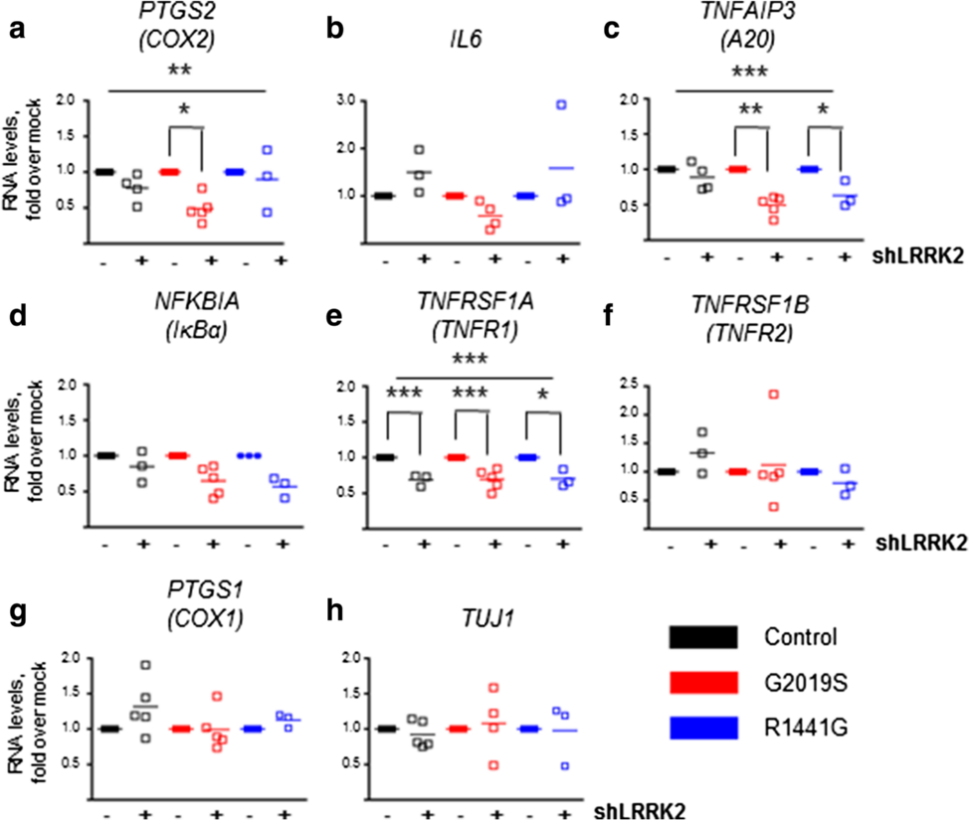
Effect of endogenous LRRK2 silencing on NF-κB target genes. **a-h** qRT-PCR analyses of inflammatory genes 5 days after LRRK2 silencing. The effect of LRRK2 silencing was evident in the regulation of RNA levels of *COX-2*, *A20*, and *TNFR1*. For *COX-2*, the effect of LRRK2 silencing was significant (two-way ANOVA, ** *P* = 0.0021,), and post hoc analyses revealed a significant effect of shLRRK2 on RNA levels specifically in LRRK2^G2019S^ neurons (** *P* < 0.01). For *A20*, in addition to the effect of knocking down the expression of LRRK2 (two-way ANOVA, *P* < 0.0001), the presence of the mutations had also a significant effect (two-way ANOVA, ** *P* = 0.008). Subsequently, there was an interaction between shLRRK2 and genotype (two-way ANOVA, ** *P* = 0.008). Indeed, post hoc analyses showed that there was an effect of LRRK2 silencing on *A20* levels in LRRK2^G2010^ (*** *P* < 0.001) and LRRK2^R1441G^ neurons (** *P* < 0.01). Finally, for *TNFR1*, the effect of LRRK2 silencing was significant in all groups (two-way ANOVA *** *P* < 0.0001; post hoc tests: *** *P* < 0.001 in Control WT and LRRK2^G2019S^ and ** *P* < 0.01 in LRRK2^R1441G^). Mock cells were transduced with the empty vector. *Lines* represent the mean ± SEM of three to five independent silencing experiments

We have previously reported a strong effect of LRRK2 silencing on NF-κB target genes in fibroblasts [6]. Therefore, we investigated the role of LRRK2 in the regulation of this pathway in mature neurons. We found that LRRK2 knockdown significantly affected *PTGS2* (*COX-2*), *TNFAIP3* (*A20*), and *TNFRSF1A* (*TNFR1*) expression, but not all to the same extent (Fig. 4a, c, e). While shLRRK2 induced a decrease in *TNFRSF1A* in all genotypes, *TNFAIP3* RNA levels were reduced only in LRRK2 mutated neurons. We did not observe significant changes in *IL-6*, *NFKBIA* (*IκBa*), and *TNFRSF1B* (*TNFR2*) expression in silenced neurons of any genotype. This indicates that LRRK2 knockdown does not affect all NF-κB target genes equally because these are additionally regulated in each specific cell context. Importantly, LRRK2 silencing did not modify the constitutively expressed genes *PTGS1* (*COX-1*) and βIII-tubulin (*TUJ1*) (Fig. 4g, h). This confirms the lack of toxicity of LRRK2 silencing in mature human neurons and the specificity of the changes observed. Taken together, these data strengthen the link between LRRK2 and the NF-κB pathway in neurons.

### TNFα effect on α-synuclein protein levels

**Fig. 5:**
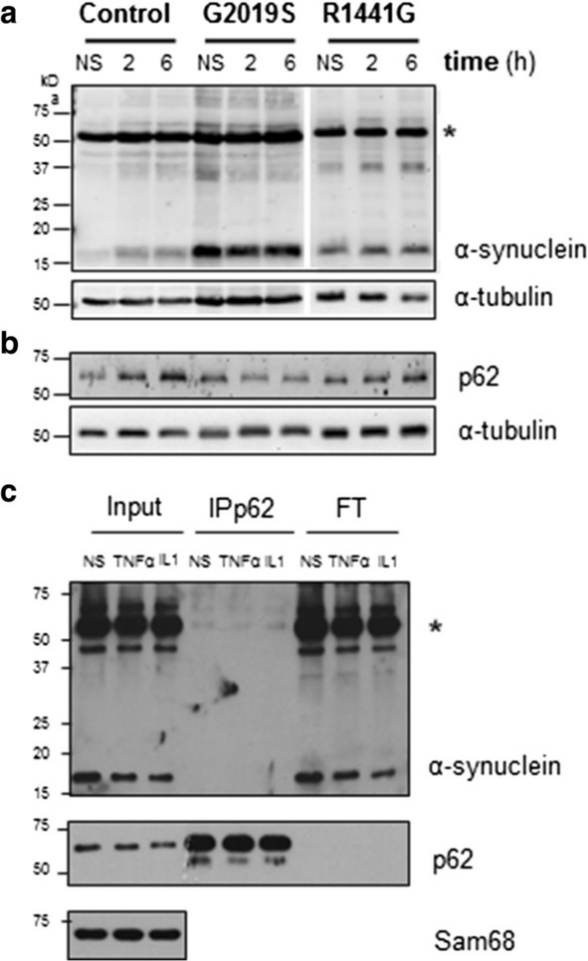
Effect of TNFα on α-synuclein. **a** Western blot analysis showing α-synuclein and **b** p62 proteins in mature neurons after treatment with TNFα, at 2 and 6 h. Proteins were resolved in 15% SDS polyacrylamide gels for the visualization of different α-synuclein oligomers. The band at 15 kDa corresponds to the monomer. A band at 50 kDa (*asterisk*) could correspond to an oligomeric form of α-synuclein. α-tubulin was used as loading control. Statistical analysis showed a significant effect of time on α-synuclein monomer levels in three to six independent experiments, including two different cell lines per genotype (RT-ANOVA, effect of time, *P* = 0.035). **c** Control WT neurons were stimulated with TNFα and with IL-1β to evaluate the interaction between p62 and α-synuclein. Co-immunoprecipitation of p62 and α-synuclein using specific antibodies did not show any association under these conditions. Sam68 was used as the loading control. *NS* non-stimulated, *IP* immunoprecipitation, *FT* flow-through. See Additional file 4 for uncropped blots

Our results so far confirmed a regulatory role of LRRK2 on α-synuclein proteostasis, as well as on NF-κB transcriptional activity. Interestingly, there are several NF-κB binding sites in the *SNCA* promoter, so we next investigated the effect of NF-κB activation with TNFα on α-synuclein levels in this paradigm. We treated mature neurons with TNFα (15 ng/ml) for the times indicated in the figure and observed a modulation of α-synuclein protein levels (Fig. 5a, Additional file 4, RT-ANOVA, effect of time, *P* < 0.05) with no significant effect of the genotype. A parallel (but not significant) trend was observed for p62 levels (Fig. 5b). The small, transient increases after TNFα challenge in all neurons support our hypothesis that inflammatory stimuli activating the NFĸB pathway can modulate α-synuclein protein levels. We next analyzed the interaction of α-synuclein with the autophagic protein p62 in WT neurons but could not detect any association between the two proteins in differentiated neurons (Fig. 5c). Thus, it is unlikely that modifications in α-synuclein in response to TNFα involve p62 in these conditions.

### Mutations in LRRK2 alter NF-κB activation

**Fig. 6:**
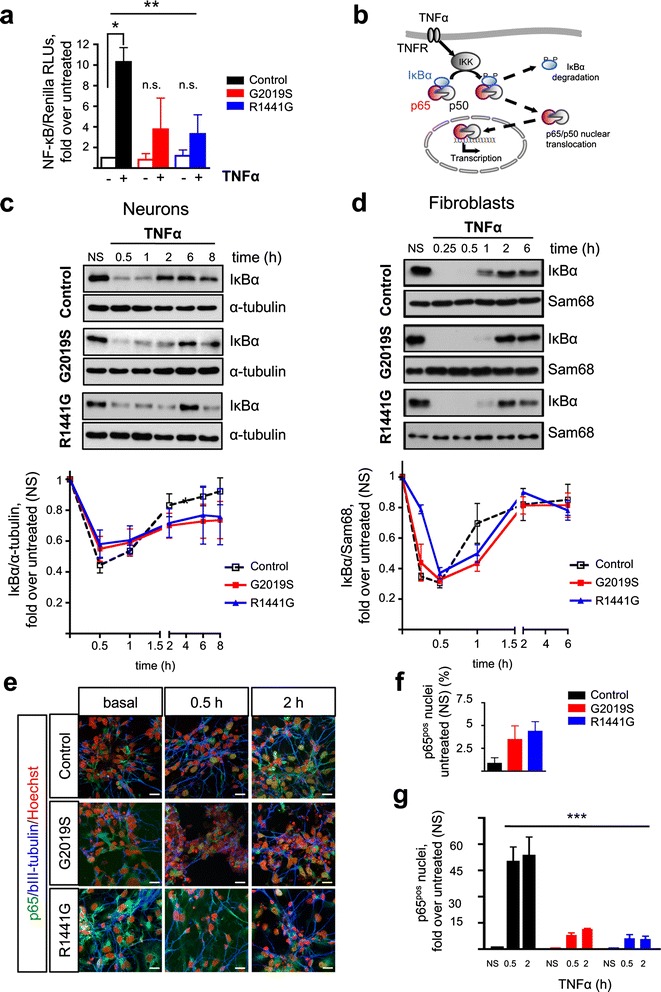
TNFα-induced NF-κB activation in neurons derived from iPSCs with LRRK2 mutations. **a** TNFα (10 ng/ml, 8 h) induced a significant NF-κB activation (two-way ANOVA, ** *P* < 0.01) but only in control WT neurons (* *P* < 0.05, post hoc test) and not in LRRK2^G2019S^ or LRRK2^R1441G^ neurons (*n.s.*). *Bars* represent the mean ± SEM of two to four determinations, including two different lines per genotype and expressed as fold values over untreated. In addition, basal NF-κB activities are normalized to the activity in the control WT neurons. **b** Schematic representation of NF-κB pathway activation. **c** Time-course of IκBα protein levels after treatment with TNFα (10 ng/ml). Immunoblots were quantified and normalized to non-stimulated (*NS*) samples. Each point in the curve is the mean ± SEM of four to five independent experiments, including two different cell lines per genotype. α-tubulin was used as the loading control. Statistical analysis showed a significant effect of time on IκBα protein levels (two-way RT-ANOVA, *** *P* < 0.0001). **d** For comparison, the same experiment is shown in fibroblasts. Points represent the mean ± SEM of three to six independent experiments, including at least two different cell lines per genotype. Sam68 was used as the loading control. Statistical analysis showed a significant effect of time on IκBα protein levels (two-way ANOVA, *** *P* < 0.0001). **e** Representative immunofluorescence staining of p65 (*green*) and βIII-tubulin (*blue*) in mature neuronal cultures incubated with TNFα (15 ng/ml, 0.5 and 2 h). Nuclei were counterstained with Hoechst 33342 (*red*). *Scale bar*, 20 μm. **f** Quantification of p65 immunoreactivity at baseline and **g** after TNFα incubation showing a significant effect of genotype (two-way ANOVA, *** *P* < 0.001). *Bars* represent the mean ± SEM of counts from two different lines per group in two to three independent experiments. *NS* non-stimulated. See Additional file 5 for uncropped blots

To examine the impact of LRRK2 mutations on NF-κB function, we treated mature neurons with TNFα (10 ng/ml, 8 h). In all the genotypes, basal NF-κB activity was similar and TNFα induced a significant NF-κB response (two-way ANOVA, *P* < 0.01). However, while the control WT cells showed a relatively large response (tenfold, *P* < 0.05, Bonferroni post hoc test), LRRK2^G2019S^ and LRRK2^R1441G^ neurons displayed a more variable activation of smaller amplitude (≤4-fold, n.s., Bonferroni post hoc test) (Fig. 6a).

Degradation of IκBα, which retains the NF-κB effector dimer p65/p50 in the cytoplasm, is an essential event for the activation of canonical NF-κB pathway following a TNFα challenge (Fig. 6b). Time-course analysis of IκBα levels showed a rapid degradation in response to TNFα in all genotypes corroborating activation of the pathway (two-way RT-ANOVA, effect of time, *P* < 0.0001). Quantification of the area under the curve (AUC) revealed a delay in the recovery of IκBα in the neurons with LRRK2 mutations (Fig. 6c, Additional file 5). During the degradation phase (0–0.5 h), the AUCs were 0.3620, 0.3874, and 0.3950 for the control WT, LRRK2^G2019S^, and LRRK2^R1441G^ neurons (C < G < R), respectively. In contrast, during the recovery phase (1–8 h), the AUCs were 5.936, 4.965, and 5.155 for the control WT, LRRK2^G2019S^, and LRRK2^R1441G^ neurons (C > R > G), respectively. Indeed, 2 h after the TNFα stimulation, IκBα levels were back to 80% of baseline in the control WT neurons while in LRRK2 mutant neurons, the levels reached only ~65% at this time. Moreover, neurons with LRRK2 mutations failed to get back to the initial values at the latest time examined (8 h). Interestingly, fibroblast cultures from these patients displayed a delayed IκBα recovery rate as well. However, in fibroblasts, IκBα levels were back to baseline in all genotypes by 2 h (Fig. 6d), suggesting that the NF-κB transcriptional defect associated with LRRK2 mutations is more pronounced in the neurons.

We next evaluated p65 nuclear translocation in mature neurons by IF at 0.5 and 2 h after TNFα stimulation (15 ng/ml). These time points were chosen to coincide with the IκBα degradation and recovery phases observed by western blot. Notably, already at baseline, LRRK2^G2019S^ and LRRK2^R1441G^ cultures had a higher percentage of cells displaying a clear nuclear p65 signal (Fig. 6e, f) (0.8 ± 0.4, 3.6 ± 1.6, and 4.7 ± 1.8 in the control WT, LRRK2^G2019S^, and LRRK2^R1441G^, respectively). TNFα induced an increase in p65 nuclear localization in the control WT cultures (50- and 53-fold over non-stimulated baseline at 0.5 and 2 h, respectively). This response to TNFα was slower and significantly attenuated in all LRRK2 mutated neurons (8 and 11.5 at 0.5 and 2 h in LRRK2^G2019S^; 6.2 and 5.8 at 0.5 and 2 h in LRRK2^R1441G^, two-way ANOVA, *F* = 24.68, *P* < 0.001) (Fig. 6e, g). These results show that neurons with LRRK2^G2019S^ and LRRK2^R1441G^ mutations have a defect in p65 translocation underlying the protracted NF-κB transcriptional response, similar to the defect that we described before in patients’ fibroblasts [6].

## Discussion

Taking advantage of iPSC technology, we investigated NF-κB signaling in patient-specific neurons harboring the G2019S and R1441G mutations in LRRK2. Some LRRK2^G2019S^, LRRK2^R1441C^, and LRRK2^I2020T^ iPSC lines have been previously established and characterized [19–27]. For this study, we derived two novel iPSC lines with the R1441G substitution, which is frequent in the Basque Country, in addition to two G2019S-iPSC lines. Reprogramming and differentiation into DA neurons was similar in all mutant lines. The efficiency of DA neuronal specification, rather limited in this study, was nonetheless comparable to that obtained from a hES cell line (H9) and to that reported in other studies [19–27]. With this small percentage of DA neurons, our results are better viewed as representative of a mature heterogeneous neuronal population. Importantly, all experiments were carried out at 6 weeks or later, when cultures mainly contained mature neurons (90% of all cells) with normal Tau expression levels and distribution. We could not detect LRRK2 during the neural induction stage (1–3 weeks), but protein expression increased at later stages following a normal developmental profile [28, 29]. LRRK2 protein levels were similar in all genotypes and, in agreement with previous reports, LRRK2 endogenous baseline expression was not increased in LRRK2^G2019S^ neurons [19, 20]. This is in stark contrast with overexpression studies that have proposed an increased dimerization and protein stability related to the mutation effect on kinase activity [25, 30]. For LRRK2^I2020T^ and LRRK2^R1441C^, the stability of the protein has been reported to be impaired [27, 31].

There is overwhelming genetic and pathological evidence for the involvement of α-synuclein in PD. Indeed, missense mutations and multiplications in *SNCA* [32–35] cause familial autosomal dominant forms of PD. α-synuclein is also the main component of the proteinaceous inclusions (Lewy bodies and neurites) considered the pathological hallmark of PD [2, 36]. Furthermore, α-synuclein propagation has been proposed to underlie disease progression in a prion-like spreading manner, although this is debatable [37]. In agreement with previous studies in iPSC-derived neurons, we found elevated levels of α-synuclein in LRRK2^G2019S^ neurons at the mature stage [18, 19, 22]. In contrast, α-synuclein was not increased in the LRRK2^R1441G^ neurons. This difference can be related to the unequal effect of the two mutations on LRRK2 kinase activity because only the G2019S mutation robustly increases it [8]. Indeed, a recent paper further supports the hypothesis of a direct link between the enhanced kinase activity in the LRRK2^G2019S^ neurons and the increase in α-synuclein levels (and subsequent formation of inclusions), as both LRRK2 specific kinase inhibitors and α-synuclein knockdown prevented inclusion formation in mutants, in vitro and in vivo [38]. In our study, LRRK2 silencing decreased α-synuclein expression in human LRRK2 mutated neurons, underscoring the tight connection between these two *PARK* gene products.

Importantly, we found that TNFα modulates α-synuclein dynamics in iPSC-derived neurons. Other studies have shown that TNFα and TLR activation in neurons increase α-synuclein levels by inhibiting autophagy [39, 40]. In this study, TNFα transiently increased α-synuclein levels, which could eventually favor protein aggregation and pathogenicity [41, 42]. Unfortunately, because of the small magnitude of the changes, inter-individual differences and technical limitations, we cannot discuss here the effect of the LRRK2 mutations on different alpha-synuclein molecular forms, which could be relevant for disease pathogenesis and deserves further work.

Wild-type LRRK2 has been proposed to activate inflammatory signaling. Overexpression of LRRK2 in vitro up-regulated the canonical NF-κB pathway [14, 16, 43, 44], while the effect of PD-associated LRRK2 mutations is less clear [14, 43]. Similarly, there is no consensus on the role of LRRK2 kinase activity on the stimulation of the NF-κB cascade [14–16, 44–46]. Discrepancies may be partly due to the use of different cellular systems and stimulation conditions and, in the case of LRRK2 inhibitors, to off-target effects. On the other hand, knockdown experiments of endogenous LRRK2 expression in primary microglia and immortalized immune cell lines down-regulated inflammatory signaling, even in the absence of a pro-inflammatory stimulus [14, 15, 44, 45], which is also in agreement with our findings in LRRK2-silenced fibroblasts [6]. In neurons, we found a differential regulation of NF-κB transcriptional targets, which may be dependent on cell-specific factors. Importantly, we could identify a significant effect on *COX-2*, validating our previous findings in fibroblasts and underscoring the preservation of the phenotype regarding the NF-κB pathway in these and other experimental models [6, 47]. From a practical point of view, this is rather convenient as dermal fibroblasts are easily accessible and expandable and can be used to screen for disease modifiers regarding this pathway. Nevertheless, neurons allowed us to explore neuronal specific proteins (such as α-synuclein) and pathways.

The low endogenous expression of LRRK2 in iPSC-derived neurons and the heterogeneous nature of the cultures resulted in relatively small TNFα-induced NF-κB response. Still, it was sufficient to detect the reduction in NF-κB transcriptional activation in response to TNFα in both LRRK2^G2019S^ and LRRK2^R1441G^ neuronal cultures. Furthermore, the recovery of IκBα protein, which is a direct transcriptional product providing feedback pathway regulation, was also impaired in mutant neurons. Given that IκBα degradation in the mutants was normal, our data pointed to a defect downstream p65 release from IκBα. Indeed, p65 nuclear translocation was defective in LRRK2 mutants. In addition, LRRK2 mutated neurons displayed slightly increased levels of nuclear p65 in the absence of any stimulus, supporting a leaky regulation of the system. These defects in iPSC-derived neurons corroborate our previous observations in patients’ fibroblasts, albeit with minor differences.

Mechanistically, our results imply that both the G2019S and R1441G mutations impair canonical NF-κB signaling at the level of p65 nuclear translocation and/or downstream transcriptional activation. Indeed, in LRRK2 mutant neurons primed with TNFα, IκBα degradation was normal like in LRRK2-silenced microglia treated with LPS [44]. p65 nuclear translocation may require LRRK2 scaffold function, perhaps through interaction with 14-3-3 proteins that could be disrupted by LRRK2 mutations [48–50]. On the other hand, mutations could alter this pathway at other levels, like the DNA-binding capacity of the transcription factor. In line with this, recent reports described higher levels of the phosphorylated form of the NF-κB inhibitory subunit p50 in LRRK2-silenced microglia, which correlated with subsequent higher p50 binding to DNA and transcriptional repression [45]. It would be very informative to characterize this response in neurons from sporadic PD patients.

## Conclusions

These results validate in neurons our previous findings in patients’ fibroblasts regarding NF-κB signaling modulation by LRRK2 and underscore the usefulness of the iPSC-neuron paradigm to study time-dependent, neuron-specific alterations in a context that retains endogenous expression of pathogenic proteins. iPSC-derived neurons carrying the G2019S and R1441G mutations in LRRK2 showed impaired canonical NF-κB signaling and altered NF-κB target gene transcription regulation upon LRRK2 knockdown. Temporal analysis following a TNFα challenge revealed a protracted recovery of IκBα protein, concomitant with defective p65 nuclear translocation in both LRRK2^G2019S^ and LRRK2^R1441G^ neurons. Although basal α-synuclein protein levels were increased in LRRK2^G2019S^ mature neurons, and not in LRRK2^R1441G^ neurons, LRRK2 silencing down-regulated α-synuclein protein expression in both. This led us to hypothesize that NF-κB and α-synuclein pathways driving PD progression might converge. Indeed, TNFα elevated α-synuclein levels, although we could not detect an effect of LRRK2 mutations. Further studies are needed to understand how long-term neuroinflammation impacts on α-synuclein dynamics and the contribution of LRRK2 mutations to this pathway.

## Additional files

Additional file 1: Table S1. Primer sequences used in RT-qPCR analyses.
Additional file 2: Uncropped blots related to Fig. 2.
Additional file 3: Uncropped blots related to Fig. 3.
Additional file 4: Uncropped blots related to Fig. 5.
Additional file 5: Uncropped blots related to Fig. 6.

## Abbreviations

DA: Dopamine
iPSC: Induced pluripotent stem cells
IκBα: Nuclear factor of kappa light polypeptide gene enhancer in B cells inhibitor, alpha
LRRK2: Leucine-rich repeat kinase *2*
NF-κB: Nuclear factor kappa-light-chain-enhancer of activated B cells
PD: Parkinson’s disease
ROC: Ras of complex proteins
SNCA: Synuclein alpha
TNF: Tumor necrosis factor
TNFR: Tumor necrosis factor receptor
WT: Wild-type
