# Supplementary material for: Mutations in LRRK2 impair NF-κB pathway in iPSC-derived neurons
Source: J Neuroinflammation. 2016 Nov 18;13:295. doi: 10.1186/s12974-016-0761-x (PMC5116223; doi:10.1186/s12974-016-0761-x)
Supplement: Additional file 1: Table S1. — Primer sequences used in RT-qPCR analyses. [file 12974_2016_761_MOESM1_ESM.pdf]

Mutations in LRRK2 impair NF-κB pathway in iPSC-derived neurons

Rakel López de Maturana, PhD[1], Valérie Lang, PhD[2], Amaia Zubiarrain[1], Amaya Sousa[1], Nerea Vazquez[1], Ana Gorostidi[3], PhD, Julio Aguila[1], PhD, Adolfo López de Munain[4,5,6], MD, PhD, Manuel Rodríguez[2], PhD, and Rosario Sánchez-Pernaute, MD, PhD[1]

Additional Material.

**Target gene**	**Forward (5'-3')**	**Reverse (5'-3')**
PTGS1 (COX-1)	CGATGCATACACCACATGAA	AGCGAAGGCTTCTCAAATCA
PTGS2 (COX-2)	GAATCATTCACCAGGCAAATTG	TCTGTACTGCGGGTGGAACA
GAPDH	TGCACCACCAACTGCTTAGC	GGCATGGACTGTGGTCATGAG
GFAP	TCTCTCGGAGTATCTGGGAACTG	TTCCCTTTCCTGTCTGAGTCTCA
IL6	ATGCAATAACCACCCCT	AGTGTCCTAACGCTCATAC
LRRK2	TCCAGATCAACCAAGGCTCACCAT	AGGCTGCTCGGTAAACTGATCCAA
MAPT	GATTGGGTCCCTGGACAATA	GTGGTCTGTCTTGGCTTTGG
NFKBIA (IκBα)	GATCCGCCAGGTGAAGG	GCAATTTCTGGCTGGTTGG
PINK1	GCTTGGGACCTCTCTTGGAT	CGAAGCCATCTTGAACACAA
SNCA	TCCAGAATTCCTTCCTGTGG	GAAGACAGTGGAGGGAGCAG
TNFAIP3 (A20)	GTCCGGAAGCTTGTGGCGCT	CCAAGTCTGTGTCCTGAACGCCC
TNFRSF1A (TNFR1)	AATGCCGAAAGGAAATGGGTCAGG	AGGTGCACACGGTGTTCTGTTTCT
TNFRSF1B (TNFR2)	TGGTGTGAAAGTCAGATGCCCAGA	TGGCAGAGTTTGGCTTTGTCGTTG
TH	TGTCTGAGGAGCCTGAGATTCG	GCTTGTCCTTGGCGTCACTG
TUJ1	AGTCGCCCACGTAGTTGC	CGCCCAGTATGAGGGAGAT

Additional Table 1. Primer sequences used in RT-qPCR analyses.
