# Supplementary figures and images for: Mutations in LRRK2 impair NF-κB pathway in iPSC-derived neurons
Source: J Neuroinflammation. 2016 Nov 18;13:295. doi: 10.1186/s12974-016-0761-x (PMC5116223; doi:10.1186/s12974-016-0761-x)

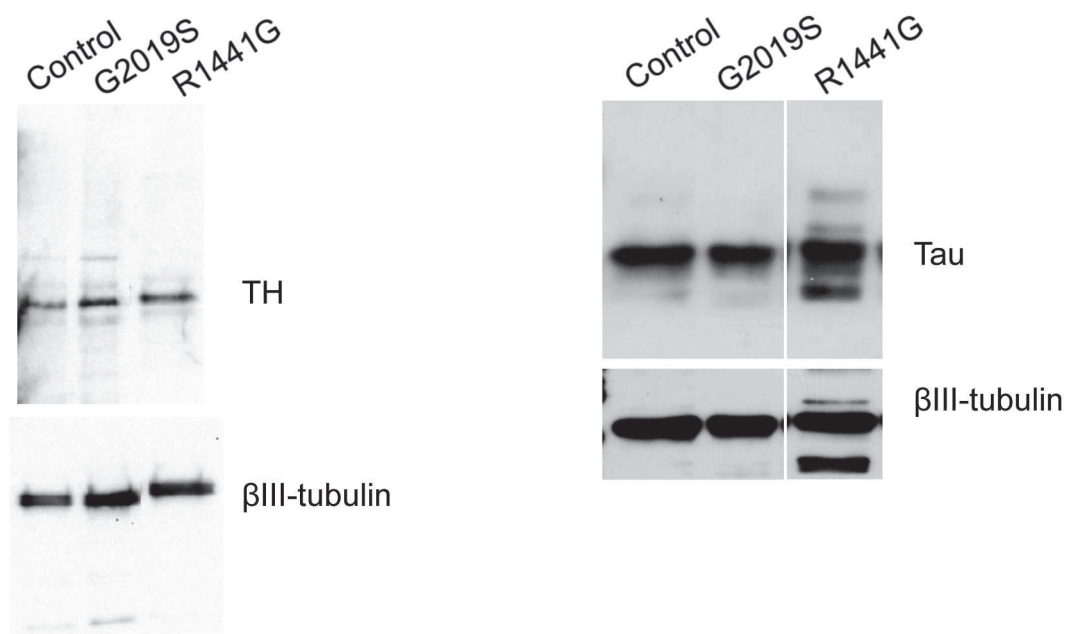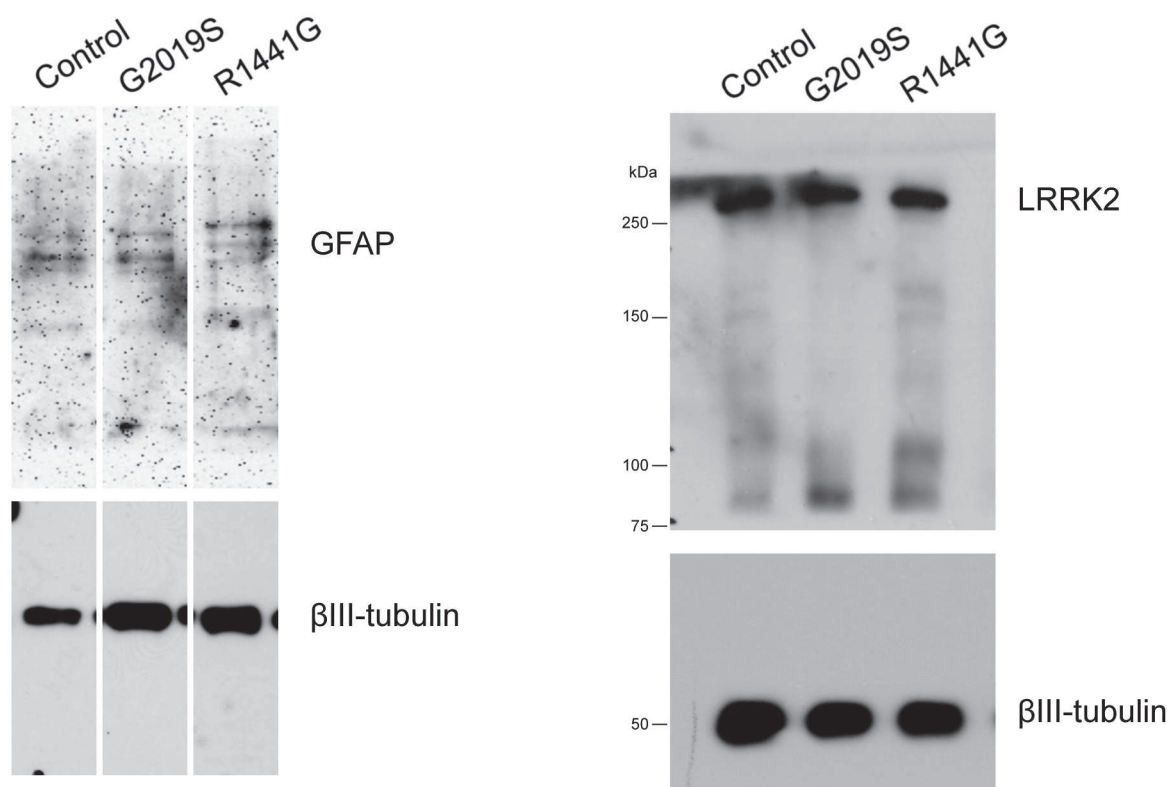

**Additional file 2.** Uncropped blots related to Figure 2.

Supplement: Additional file 2: — Uncropped blots related to Fig. 2. [file 12974_2016_761_MOESM2_ESM.pdf]

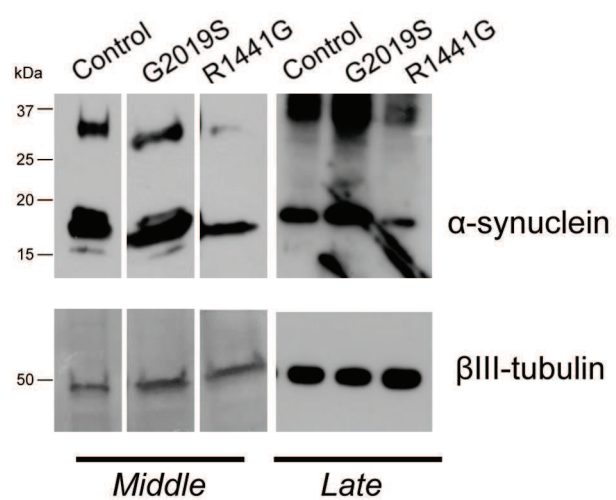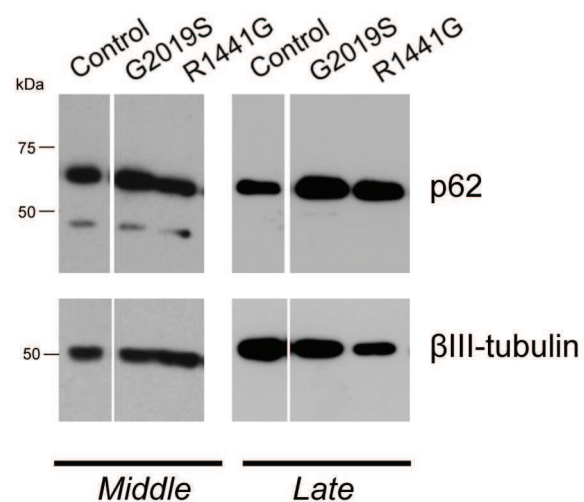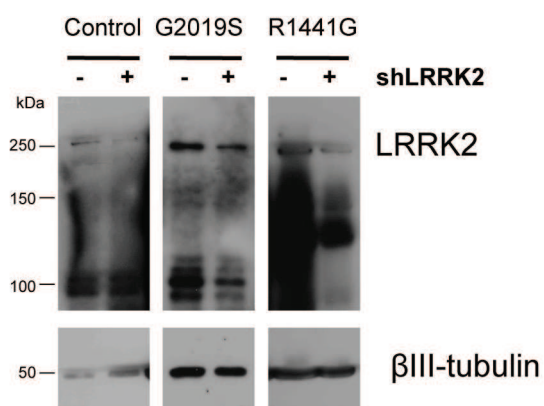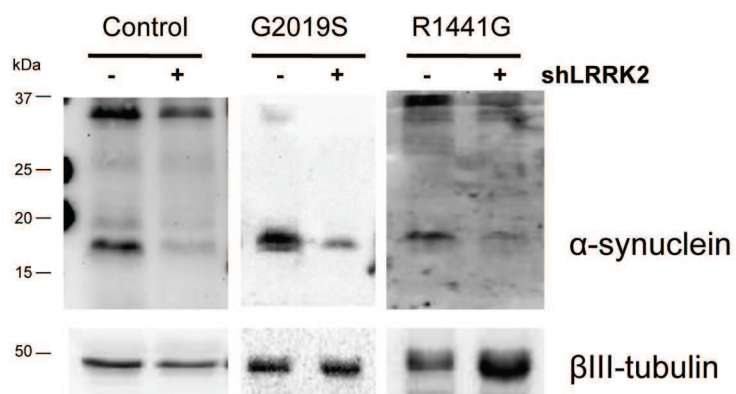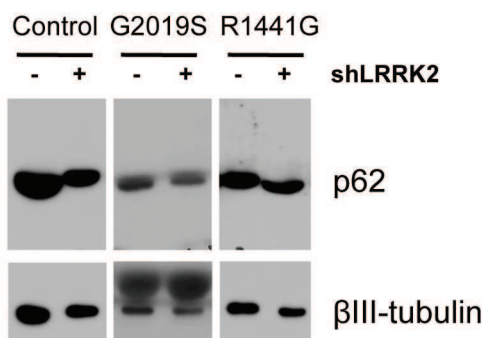

**Additional file 3.** Uncropped blots related to Figure 3.

Supplement: Additional file 3: — Uncropped blots related to Fig. 3. [file 12974_2016_761_MOESM3_ESM.pdf]

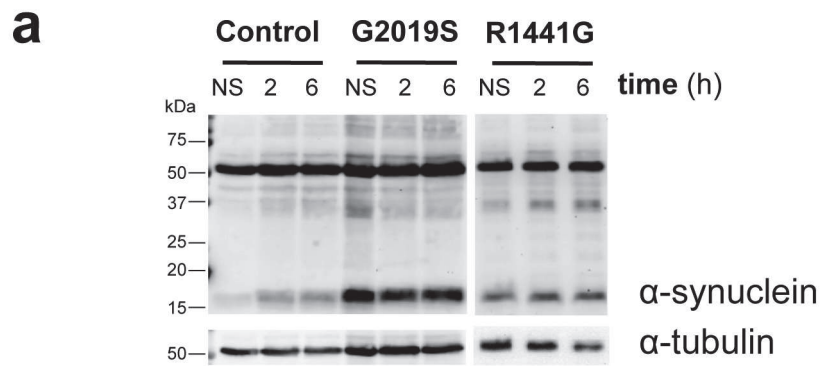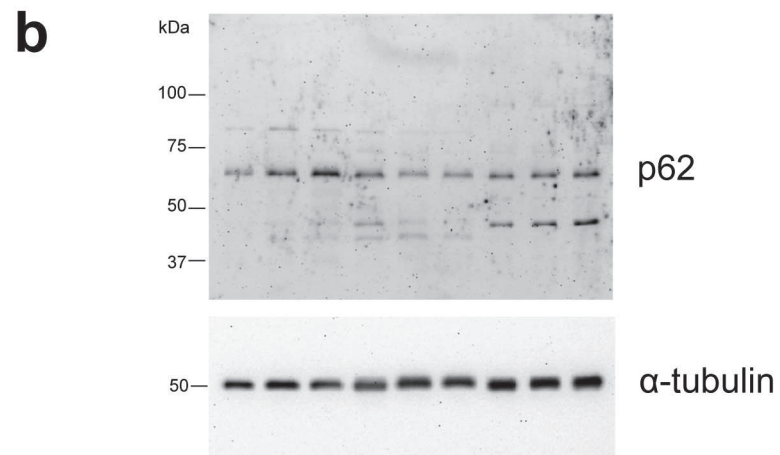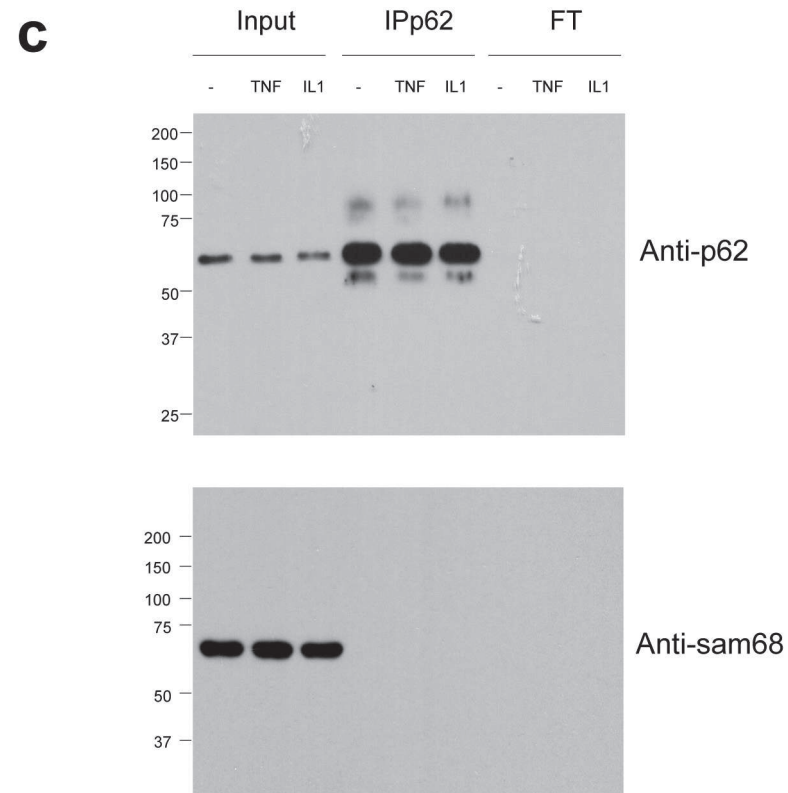

Supplement: Additional file 4: — Uncropped blots related to Fig. 5. [file 12974_2016_761_MOESM4_ESM.pdf]

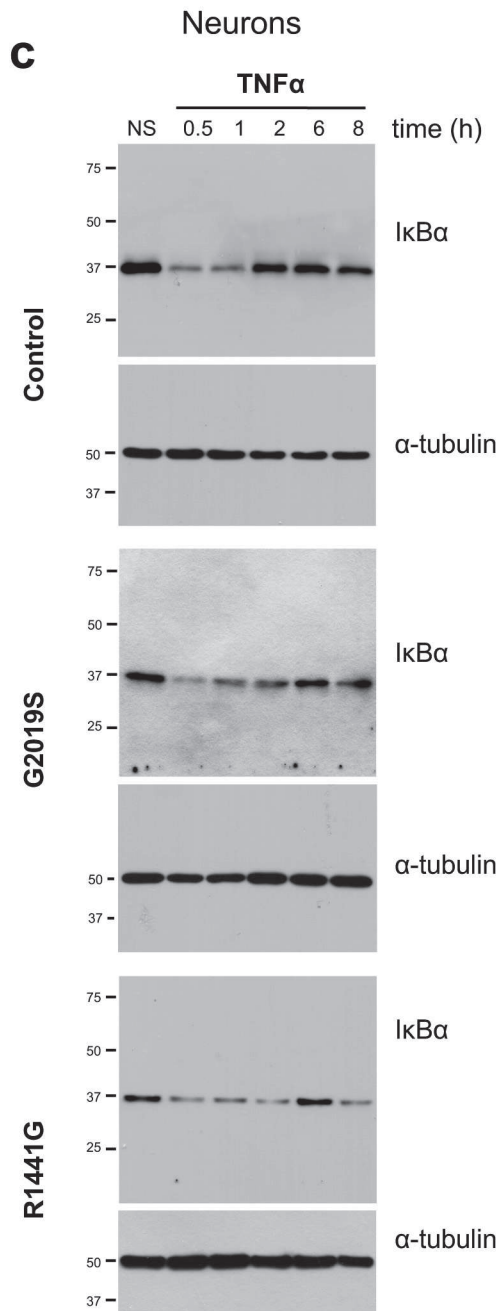

**Additional file 5.** Uncropped blots related to Figure 6

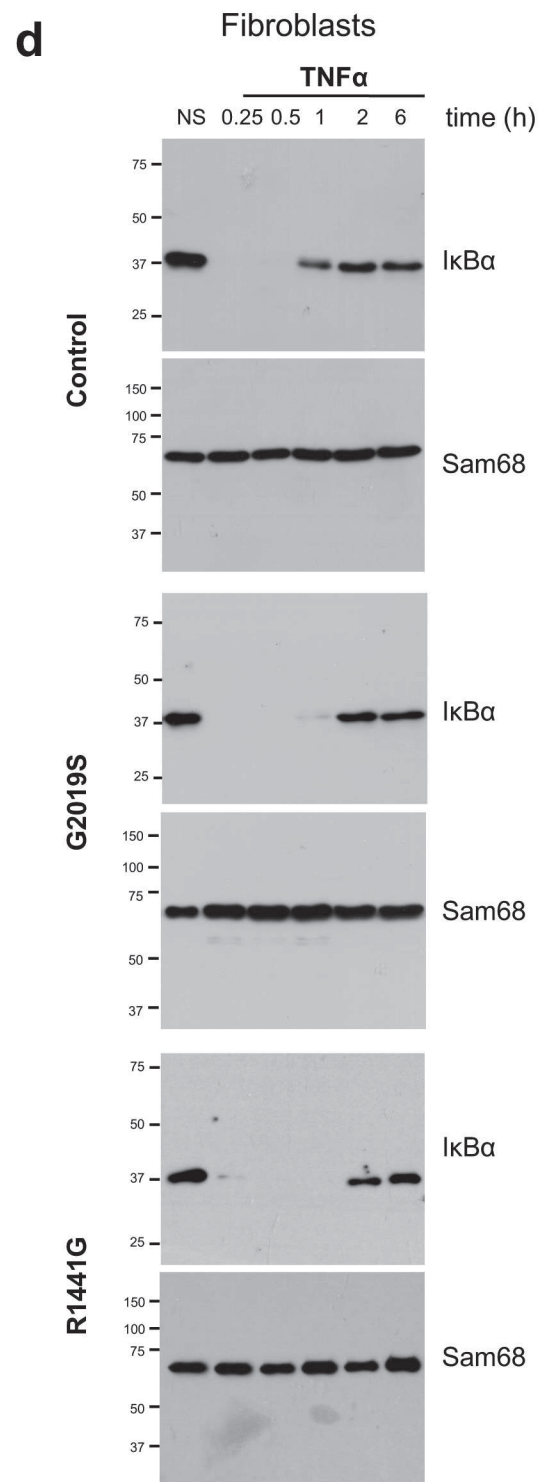

Supplement: Additional file 5: — Uncropped blots related to Fig. 6. [file 12974_2016_761_MOESM5_ESM.pdf]
